# Effect of education and health locus of control on safe use of pesticides: a cross sectional random study

**DOI:** 10.1186/1745-6673-7-3

**Published:** 2012-02-25

**Authors:** Sherine Gaber, Soha Hassan Abdel-Latif

**Affiliations:** 1 Health Education and Behavioral Sciences, High Institute of Public Health, Alexandria University, Alexandria, Egypt; 2 Vector Control and pesticides risks, High Institute of Public Health, Alexandria University, Alexandria, Egypt

**Keywords:** Pesticide use, Knowledge, Behavior, Health locus of control, Egypt

## Abstract

**Background:**

In Egypt, many pesticides are used to control pests in agricultural farms. Our study aimed to investigate knowledge and behaviors of farmers related to pesticide use and their relation to educational level and health locus of control. Health locus of control is the degree to which individuals believe that their health is controlled by internal or external factors.

**Methods:**

A cross-sectional randomized approach was used to collect data from 335 farmers in Mahmoudiya region, Egypt using an interview questionnaire. Results were analyzed using Pearson Chi-square test, Fisher's exact test, Student *t*-test and ANOVA.

**Results:**

The average age of farmers was 34 years and 61% of them didn't receive school education. School education was related to higher levels of knowledge and behaviors. Farmers who received school education had more knowledge about the negative effects of pesticides on health and routes of contamination with pesticides. They also had higher scores on reading labels of pesticides containers and taking precautions after coming in contact with pesticides. Regarding health locus of control, higher internal beliefs were significantly related to higher knowledge and behaviors scores, while there was no significant relation between chance and powerful others beliefs with knowledge or behaviors.

**Conclusion:**

In the present study, higher level of education and lower level of internal beliefs were related to better knowledge and safer use of pesticides among Egyptian farmers. We recommend that strategies for raising internal beliefs must be included in health education programs that aim to ameliorate pesticides use among farmers.

## Background

In Egypt, several pesticides including organophosphorus, carbamate, pyrethroid insecticides, fungicides, and herbicides are commonly used to increase agricultural productivity [1]. Pesticides have serious drawbacks on human health as they affect the immune system, the endocrine system and the nervous system [2]. Studies conducted in Egypt found that organophophorus and carbamate insecticides increase the risk of developing hepatocellular carcinoma in farmers having HCV and HBV infection [3]. Lack of following safety measures among Egyptian farmers has many reasons: illiteracy, unavailable protective devices, low awareness about the danger of pesticide contamination and the neglect of legislation regulating pesticide use [4,5].

Farmers' knowledge and perception about pesticide risks play an important role in determining the use of pesticides protection devices (PPD) [6]. Education level has an important role in increasing knowledge about pesticides' risks [7,8]. However, knowledge is not sufficient if farmers have low confidence in their ability to apply safety measures related to pesticides use [9]. Health locus of control, which is the degree to which individuals believe that their health is controlled by internal or external factors, can be one of the explanation of farmers unsafe behaviors related to pesticides [10]. Health Locus of Control (HLC) theory categorize individuals into "health-externals" and "health-internals." External locus of control is the belief that our fate is controlled by external forces as other people or luck. Internal locus of control is the belief that our destiny is determined by our behaviors [11].

The aim of the research was to determine the relation between education and health locus of control to knowledge and behaviors of farmers to pesticide use in Mahmoudiya, Behera Governorate, Egypt.

## Methods

### Study setting

The study was conducted in the main Family Health Center in Mahmoudyia city, the capital of Mahmoudyia region, Behera governorate, Egypt. This region was selected because agriculture is the main occupation in the area and different types of pesticides are used due to the diversity of crops such as rice, fruits and vegetables.

### Study design and target population

A cross sectional random study was used to conduct the research. As pesticide spraying is a male job all over Egypt, male farm workers attending the Family Health Center were randomly selected to participate in the study (every fourth patient). 335 farmers were willing to participate with a response rate of 36.6%. 68% of non-respondents were above 50 years old, with no difference between respondents and non-respondents regarding level of education or income.

### Questionnaire design

After review of literature [6,9,12-16] an interview questionnaire was designed to assess knowledge, behaviors of participants to pesticide use and their health locus of control. A pilot study was conducted with 15 farmers to test the questionnaire revealing that the six-point likert scale of health locus of control was too difficult for the farmers as they couldn't differentiate between slightly/moderately disagree and slightly/moderately agree so a four-point likert scale was used (strongly disagree, disagree, agree, strongly agree).

The interview was conducted by the researchers and two nurses trained on filling the questionnaire. The questionnaire included personal and family data as: age, marital status, educational level, income (poor: below 2$ dollars poverty line), medical history of chronic diseases and the type of pesticides commonly used.

Knowledge questions included 10 items about acute and chronic toxicity of pesticides, health hazards of different pesticides, pesticide absorption through skin, the relation between pesticide concentration and health hazards, appropriate clothes used during spraying, the effect of pesticide leakage to underground water and re-entry of field following pesticide spraying. The answers were scored so that wrong answer scored 0, "don't know" scored 1 and right answer scored 2 with a total score of 20.

Behavior questions included 11 items focusing on methods of protection during mixing and spraying of pesticides, use of protective devices, washing and taking a bath after application, clothing (used during spraying, changing and separating clothing following spraying), eating and drinking during pesticide application, cleaning nozzle, re-entry period in the farm after applying pesticides, disposal of pesticide container. Wrong behavior was scored 0 while good behavior was scored 1 with a maximum total score of 11.

Multidimensional Health Locus of control Scale (Form A) was used to determine the participants' health locus of control [11].

### Statistical analysis

Data were collected, coded, analyzed, and tabulated using the Statistical Package for Social Sciences (SPSS Inc., Chicago, IL, USA) Version 19. The Pearson Chi-square test and Fisher's exact test were used to compare the categorical data. Student *t*-test and ANOVA test were used the quantitative data. Level of significance was determined at *P*-value ≤ 0.05.

### Ethical considerations

A verbal approval was obtained from the participants after describing the aim of the study. The Research was approved by a panel of professors in health education and vector control departments in the High Institute of Public Health, Alexandria University.

## Results

Table (1) shows that the sample included 335 farm employees. The mean age of the farmers was 33.9 ± 11.516 (range 15-62 years). Most of them (61%) had no school education and (75%) were considered poor. (35%) of the sample had chronic diseases of whom 9.6% and 6.6% suffered from cardiac and respiratory diseases respectively. 4% of farmers had a history of acute poisoning with pesticides.

**Table 1:** Sociodemographic and health conditions of the study sample

	Number	Percent
**Age **(mean)		

**Marital status**		

Single	77	23

Married	231	69

Divorced/widow	27	8

**Education**		

No school education	204	61

School education	131	39

**Income**		

Poor	251	75

Not poor	84	25

**Chronic disease**		

Present	117	35

Absent	218	65

**History of acute pesticide poisoning**		

Present	14	4

Absent	321	96

In Table (2), the results show that the percent of correct answers among the whole sample was high about ability of pesticides to reach underground water (93%), negative effect of pesticides on health (82%) and the effect of higher concentration of pesticides on health (65%). The percent of correct answers was low about: wearing long sleeves and trousers during spraying (5%), re-entry of field following pesticide application (5%) and long negative effects of pesticides (14%). The highest percent of "don't know" answers were related to staying away from the field after pesticide application (73%) and wearing long sleeves during application (59%). While 35% of farmers had wrong belief that all pesticides have same health hazards and 27% of them believed that pesticides can't be absorbed through skin.

**Table 2:** Effect of educational level on knowledge of farmers about pesticides

Knowledge (correct answer)	Correct answer		Correct answer of total sample N=335	*χ*^2^
	**No school education N=204**	**School education N=131**		

Pesticides have negative effect on health	72%	97%	82%	34.06**

All pesticides have same negative effects on health	15%	67%	35%	96.25**

Long term negative effects of pesticides	4%	30%	14%	41.79**

Symptoms of acute poisoning with pesticides	21%	44%	30%	19.17**

Eating, drinking and smoking in the field increased pesticide toxicity	22%	48%	32%	25.81**

Pesticides can be absorbed through skin	9%	61%	29%	105.21**

Higher concentration of pesticides leads to increase rate of poisoning	48%	91%	65%	64.04**

Re-entry of field following pesticide spraying	3%	9%	5%	6.06**

Wearing long sleeves and trousers during pesticide application	2%	9%	5%	9.09**

Pesticide can reach underground water	89%	100%	93%	15.12**

**Total mean score**	9.54 ± 2.2	14.20 ± 2.5**	11.36 ± 3.2	17.56**

**p ≤ 0.05

Regarding the relation between knowledge and educational level, the table shows that farmers who received school education had higher levels of knowledge than those who did not. This difference was most obvious regarding knowledge about absorption of pesticides through skin(61% versus 9%), the effect of different types of pesticides on health (67% versus 15%), long term negative effects of pesticides (30% versus 4%) and the effect of high concentration of pesticides on health (91% versus 48%). The smallest difference in knowledge between the two groups was about: Wearing long sleeves and trousers during pesticide application. The difference in total mean score was significant between the two groups.

Table (3) shows that the majority of the farmers drinks and eats during spraying (100% and 99% respectively), 100% of them neither wear a protective uniform nor use special clothing during pesticide application and 98% of the farmers don't put a mask during spraying. Only 26% of the sample takes a bath and 10% change their clothes following pesticides application.

**Table 3:** Effect of education level on behaviors related to pesticides use

Behavior	No school education	School education	Total	*χ*^2^
**Read labels on pesticide container**				
No	81%	44%	67%	51.38**
Yes	19%	56%	33%	

**Mix pesticides with water**				
Hand covered with cloth or plastic bag	54%	21%	42%	35.86**
Using gloves	46%	79%	58%	

**Clean sprayer nozzle**				
Blowing by mouth	61%	34%	50%	24.46**
Using wire	39%	66%	50%	

**Pesticide comes in contact with the body**				
Do nothing	80%	47%	67%	38.38**
Washing site of contact	20%	53%	33%	

**Protect himself during spraying**				
Just take care	100%	100%	100%	
Wearing protective uniform	0%	0%	0%	

**Put mask during spraying**				
No	99%	98%	98%	1.93
Yes	1%	2%	2%	

**Wear special clothes during spraying**				
Wearing everyday clothes	100%	100%	100%	
Have special clothes for spraying	0%	0%	0%	

**Wash hands and face after pesticide application**				
No	89%	77%	42%	33.19**
Yes	11%	23%	58%	

**Take a bath following pesticide application**				
No	78%	68%	74%	8.09**
Yes	22%	32%	26%	

**Change clothing after pesticide application**				
No	92%	87%	90%	2.36
Yes	8%	13%	10%	

**Wash clothes used during pesticide application separately**				
No	96%	99%	97%	1.58
Yes	4%	1%	3%	

**Drink during pesticide application**				
No	0%	0%	0%	
Yes	100%	100%	100%	

**Dispose pesticide container**				
Used in house	53%	28%	43%	19.52**
Disposed with usual trash	45%	66%	53%	
Burned	2%	6%	4%	

**Total (mean score)**	2.72 ± 1.79	4.97 ± 2.12	3.60	

The results show that participants who received school education had higher percent of healthy behavior than those who did not receive any school education. They had a higher percentage regarding: reading labels on pesticides containers, mixing pesticides using gloves, cleaning sprayer nozzle using a wire, washing skin coming in contact with pesticides, putting a cloth on nose and mouth during spraying, washing hands and face and taking a bath following pesticide application and they had a low percentage of using pesticide containers at home. While there was no significant difference between those receiving school education and those who did not regarding wearing protective uniform or special clothes during spraying, changing and washing clothing after pesticide application and eating or drinking during pesticide spraying.

There was a significant difference between the two groups in the total mean score of knowledge.

**Figure 1:**
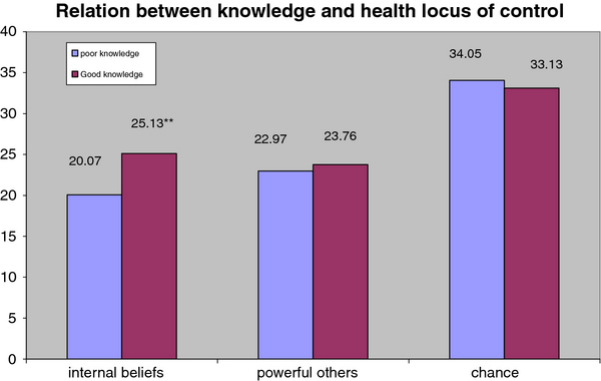
**Relation between knowledge and health locus of control**.

Figure 1 shows the relation between knowledge and health locus of control. There was a significant statistical relation between internal beliefs and knowledge with good knowledge related to higher internal beliefs. On the other hand, there was no significant relation between knowledge and either beliefs about powerful others or chance beliefs. The total sample had higher chance beliefs than internal beliefs. Younger age group (15-32 years old) had higher internal beliefs and lower beliefs in powerful others (t = 9.771 and 3.969 respectively, *p *≤ 0.05.). School education and sufficient income were also significantly related to higher internal beliefs (t = 11.520 and 4.178 respectively, *p *≤ 0.05)

**Figure 2:**
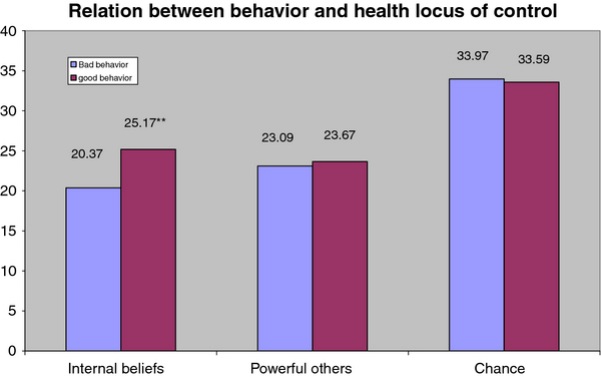
**Relation between behaviors related to pesticide use and health locus of control**.

Figure 2 shows the relation between behaviors and health locus of control. There was a significant statistical relation between internal beliefs and behaviors related to pesticides use (t = 7.842, *p *≤ 0.05.) with good behavior related to higher internal beliefs while there was no significant relation between behaviors and either beliefs about powerful others or chance beliefs.

## Discussion

In African countries, many governments encourage the use of pesticides [17]. Protective measures should be followed by farm workers to protect themselves from contamination during handling pesticides [18]. In this study, 335 farmers were interviewed to assess the effect of their educational level and health locus of control on their knowledge and behaviors about pesticides use.

The results show that among the total sample, the highest level of correct answers was about the ability of pesticides to reach underground water followed by awareness of negative effect of pesticides on health (93% and 82% respectively) which is contradictory to the results of a study conducted in Gaza where farmers had low awareness about the ability of pesticides to reach groundwater [19]. On the other hand, the lowest percent of correct answers was about wearing long sleeves during spraying and time of re-entry to the field.

Higher education was significantly related to higher percent of correct answers in all aspects of knowledge. The difference was specially noticed in knowledge about absorption of pesticides through skin and all pesticides have not same health effects. These results confirmed the findings of the earlier studies indicating that there are a significant relation between farmers' educational level and their level of knowledge [19,20].

Regarding behaviors related to pesticides use, most of the farmers (67%) did not read labels or instructions on the pesticide containers. A study conducted in Ethiopia found most of farmers didn't read instructions on pesticides packages due to illiteracy or they are just reluctant to read them [6].

The results show that 100% of farmers didn't use PPD which is consistent with the results of many studies conducted in many parts of the world [6,13-15,21,22] and another agricultural areas in Egypt [16,23]. The reasons for not using PPD among the present sample could be due to low level of knowledge about the safety measures, unavailability of protective devices at governmental agricultural association and their high cost at private sector. Hot weather was among the causes of low use of PDD as reported by studies conducted in USA [24].

Most of the farmers did not take a shower or change their clothes after pesticide application and did not wash their clothes used during pesticide application separately. The attitude of the workers towards hygiene and sanitation must be improved, and there is scope for the provision of better facilities and infrastructures. The majority of farmers disposed pesticide containers by using them at home or discarding them with usual trash which is consistent with the results of two studies conducted in Greece and in Gaza [19,25]. In contradiction, a study conducted in Iran reported that more than half of the farmers sell empty containers for recycling, farmers in the present sample had no notion about recycling [20].

The percent of safe behaviors was higher among farmers who received school education which can be explained by higher level of knowledge. This is consistent with studies reporting that low education level limits the ability of farmers to fully understand all the health risks of pesticides and the importance of safety measures [19,26]. Most of the sprayers regardless their educational level ate and drank during pesticide work. Similar behavior was reported in other developing countries [27,28].

Regarding health locus of control, results reveal that the mean score of chance beliefs was much higher than internal or powerful others beliefs. These finding is similar to the results of studies conducted in USA [29,30] where farm workers had externality scores higher than internality related to pesticide use and farm accidents.

In the present study, young farmers, farmers who received school education and those with sufficient income scored higher in internal beliefs. The differences in health locus of control scores across educational levels is consistent with previous studies [29,31] which indicate that older age and those with less education reported higher external than internal beliefs. Health locus of control may be a step in the pathway between socio-economic status and health [32].

Farmers with higher level of knowledge and behaviors scored higher in internal beliefs which indicate that perceived internal or external control influenced individuals' confidence about their skills and planning ability [32]. This finding is supported by the study of Vela Acosta et al. [29]. where farm workers who had higher internal beliefs showed better pesticide knowledge and higher intention to improve their behaviors regarding safe use of pesticides.

The study has certain limitation as the response rate was 37% which may restrict the generalization of the results. However, as non- respondents were above 50 years old and there was no educational difference between them and respondents, it is doubtful that they had a safer pesticides behaviors.

## Conclusion

We can conclude from the study that farmers in Mahmodyia region are in need for health education programs to provide them with detailed instructions about precautions that must be taken during mixing & spraying of pesticides and introduce them to the principle of recycling. Health education programs must incorporate strategies to raise internal beliefs of farmers as persons with high internal locus of control are more likely to adopt safety behaviors. Providing PDD for farmers with reasonable prices is another measure than can be taken to encourage them to take safety precautions. Used containers can be collected for reduced prices to decrease inappropriate disposition.

## Competing interests

The authors declare that they have no competing interests.

## Authors' contributions

HSG and A-LSH contributed in the design of the study and collection of data. HSG performed the data analysis and drafted the manuscript and A-LSH reviewed the manuscript and checked for accuracy of analysis. All authors read and approved the final manuscript.
